# Feasibility of Doppler Ultrasound for Cortical Cerebral Blood Flow Velocity Monitoring During Major Non-cardiac Surgery of Newborns

**DOI:** 10.3389/fped.2021.656806

**Published:** 2021-03-22

**Authors:** Sophie A. Costerus, Anna J. Kortenbout, Hendrik J. Vos, Paul Govaert, Dick Tibboel, René M. H. Wijnen, Nico de Jong, Johan G. Bosch, Jurgen C. de Graaff

**Affiliations:** ^1^Department of Paediatric Surgery, Erasmus MC University Medical Centre-Sophia Children's Hospital, Rotterdam, Netherlands; ^2^Department of Biomedical Engineering, Thorax Centre, Erasmus MC University Medical Centre, Rotterdam, Netherlands; ^3^Department of Neonatology, Ziekenhuis Netwerk Antwerp, Middelheim Antwerp, Belgium; ^4^Department of Anaesthesiology, Erasmus MC University Medical Centre, Rotterdam, Netherlands

**Keywords:** cerebral blood flow velocity, Doppler ultrasound, monitoring, newborns, pediatric anesthesia, perioperative management

## Abstract

**Background and Aim:** Newborns needing major surgical intervention are at risk of brain injury and impaired neurodevelopment later in life. Disturbance of cerebral perfusion might be an underlying factor. This study investigates the feasibility of serial transfontanellar ultrasound measurements of the pial arteries during neonatal surgery, and whether perioperative changes in cerebral perfusion can be observed and related to changes in the perioperative management.

**Methods:** In this prospective, observational feasibility study, neonates with congenital diaphragmatic hernia and esophageal atresia scheduled for surgical treatment within the first 28 days of life were eligible for inclusion. We performed transfontanellar directional power Doppler and pulsed wave Doppler ultrasound during major high-risk non-cardiac neonatal surgery. Pial arteries were of interest for the measurements. Extracted Doppler ultrasound parameters were: peak systolic velocity, end diastolic velocity, the resistivity index and pulsatility index.

**Results:** In 10 out of 14 patients it was possible to perform perioperative measurements; the others failed for logistic and technical reasons. In 6 out of 10 patients, it was feasible to perform serial intraoperative transfontanellar ultrasound measurements with directional power Doppler and pulsed wave Doppler of the same pial artery during neonatal surgery. Median peak systolic velocity was ranging between 5.7 and 7.0 cm s^−1^ and end diastolic velocity between 1.9 and 3.2 cm s^−1^. In patients with a vasoactive-inotropic score below 12 the trend of peak systolic velocity and end diastolic velocity corresponded with the mean arterial blood pressure trend.

**Conclusion:** Perioperative transfontanellar ultrasound Doppler measurements of the pial arteries are feasible and provide new longitudinal data about perioperative cortical cerebral blood flow velocity.

**Trial Registration:** https://www.trialregister.nl/trial/6972, identifier: NL6972.

## Introduction

Newborns requiring major surgical intervention are at risk for brain injury and impaired neurodevelopment later in life (1–3). A review including a meta-analysis showed this applies to all sorts of non-cardiac congenital anomalies (3). A study in 101 neonates who had an MRI exam 7 days after different types of non-cardiac major surgical procedures reported an incidence of brain injury of 75% in preterm and 58% in term neonates (2). In here, parenchymal lesions (punctate white matter lesions, punctate cerebellar lesions, thalamic infarction, periventricular hemorrhagic infarction) were found in 42% and non-parenchymal lesions (supra- and infratentorial subdural hemorrhages, intraventricular hemorrhage grade II, asymptomatic sinovenous thrombosis) in 38% of the term neonates. A case series (*n* = 6) of infants with severe post-operative encephalopathy showed supratentorial watershed infarction in the border zone between the anterior, middle, and posterior cerebral arteries after relatively moderate surgical procedures (4). Disturbance of cerebral perfusion might be the underlying factor triggering brain injury (4, 5).

Despite state-of-the-art care, anesthetic monitoring lacks sensitivity to detect altered cerebral perfusion during high-risk neonatal surgery (6). Transfontanellar ultrasound with directional power Doppler (DPD) and pulsed wave Doppler (PWD) allows imaging real-time cerebral perfusion and quantifying cortical cerebral blood flow velocity (CBFV) in large arteries or veins. PWD is used for measuring flow velocities by calculating the Doppler waveform shift, while DPD presents the amount of Doppler energy from the vessel, instead of an alteration in velocity and therefore represents the amount of blood flow (perfusion). Doppler ultrasound is particularly feasible in neonates because of the open anterior fontanel, and is frequently used in the neonatal intensive care to screen for intracranial hemorrhages or thrombosis (7). Recently measuring cortical CBFV has been introduced during neonatal cardiac surgery under cardiopulmonary bypass (8).

Transfontanellar ultrasound with DPD and PWD might provide insight in the perioperative cerebral cortical blood flow in neonates, which is currently a black box. So far, one study used transfontanellar ultrasound intraoperatively, although they measured the internal carotid artery to predict fluid responsiveness in a specific patient group following cardiac surgery and a mean age of 5 months (9). In view of the above mentioned brain injuries, we consider the cortical arteries within the supratentorial watershed area of interest for creating understanding about the trigger for perioperative brain injury.

The aim of this study was to determine the feasibility of perioperative serial transfontanellar ultrasound measurements of the pial artery with DPD and PWD in neonates undergoing high risk non-cardiac surgery. Additionally, we investigate whether inter- and intra-individual perioperative changes in cortical CBFV can be observed with DPD/PWD and can be related to changes in perioperative management which might indicate the additional value of this new technique in comparison to conventional monitoring systems.

## Materials and Methods

In this prospective, observational feasibility study, we performed transfontanellar ultrasound with DPD and PWD before, during and after surgical treatment of neonates with 2 major congenital anomalies: congenital diaphragmatic hernia (CDH) or esophageal atresia (EA). Measurements were performed after institutional research board approval and written informed consent from both parents. Ethical approval was provided by the Medical Ethical Committee of Erasmus Medical Center, Rotterdam, the Netherlands on 13 February 2019 (Chairpersons Professor H. J. Metselaar and Professor H. W. Titanus, protocol number MEC 2017-145, amendment feasibility study, trial registration NL6972, URL: https://www.trialregister.nl/ trial/6972).

### Patients

Children born with CDH or EA scheduled for surgical repair between May 2019 and February 2020 were eligible for inclusion, except those with chromosomal anomalies, syndromes associated with major cognitive impairment or complex cardiac anomalies. CDH neonates were managed according to the CDH-EURO consortium protocol (10). EA neonates breathed spontaneously post-natally and received respiratory support if necessary.

We focused on patients with CDH or EA representing major non-cardiac anomalies that require surgical repair within the first days of life. Patients with CDH are cardiopulmonary unstable due to the lung hypoplasia and pulmonary hypertension, while neonates with an isolated EA are cardiopulmonary stable.

### Data Collection

Cerebral perfusion is quantified as a volume of flow per unit time and is affected by perioperative management, fluctuations in PaO_2_ and PaCO_2_, vaso-active medication and changes in blood volume (11). Therefore, the following data were obtained: continuous registration of vital parameters (heart rate, blood pressure, saturation) (Primus, Draeger, Luebeck, Germany), cerebral regional oxygenation (rSO_2_) with neonatal sensor (INVOS 5100C, Covidien, Boulder, Colorado, United States), and continuous transcutaneous carbon dioxide levels (Sentec, Therwil, Switzerland) were registered and analyzed off-line. Continuous transcutaneous pCO_2_ measurements were used in combination with intermittent arterial blood gas analyses. Ventilation settings, medication, fluids, and arterial blood gas analysis were registered in the electronic patient record (Hix, Chipsoft, Amsterdam, the Netherlands). Patients received general anesthesia with sevoflurane (end expiratory concentration: 1–2%) in the operation theater or bolus complemented with continuous dosage of midazolam (200 μg kg^−1^ h^−1^) if surgery was performed in the pediatric intensive care unit (PICU). Both groups also received fentanyl (1.5–3 μg kg^−1^) and rocuronium (0.5–1 mg kg^−1^). After induction of anesthesia, patients with CHD or EA planned for thoracoscopic surgery were positioned laterally and for open surgery in supine position.

The vasoactive-inotropic score (VIS) was calculated to quantify necessity of cardiovascular support (12). VIS reflects the grade of vasoactive/inotropic pharmaceutical intervention and is calculated: dopamine dosage (μg kg^−1^ minute^−1^) + dobutamine dosage (μg kg^−1^ minute^−1^) + 100x epinephrine dosage (μg kg^−1^ minute^−1^) + 100x norepinephrine dosage (μg kg^−1^ minute^−1^) + 10x milrinone dosage (μg kg^−1^ minute^−1^) + 1,000x vasopressin dosage (U kg^−1^ minute^−1^). Vital parameters and VIS were analyzed off-line from electronic registration obtained during successful ultrasound measurement.

Routine preoperative and post-operative cranial ultrasound scans were performed by an experienced pediatric radiologist to screen for cerebral injury.

**Figure 1:**
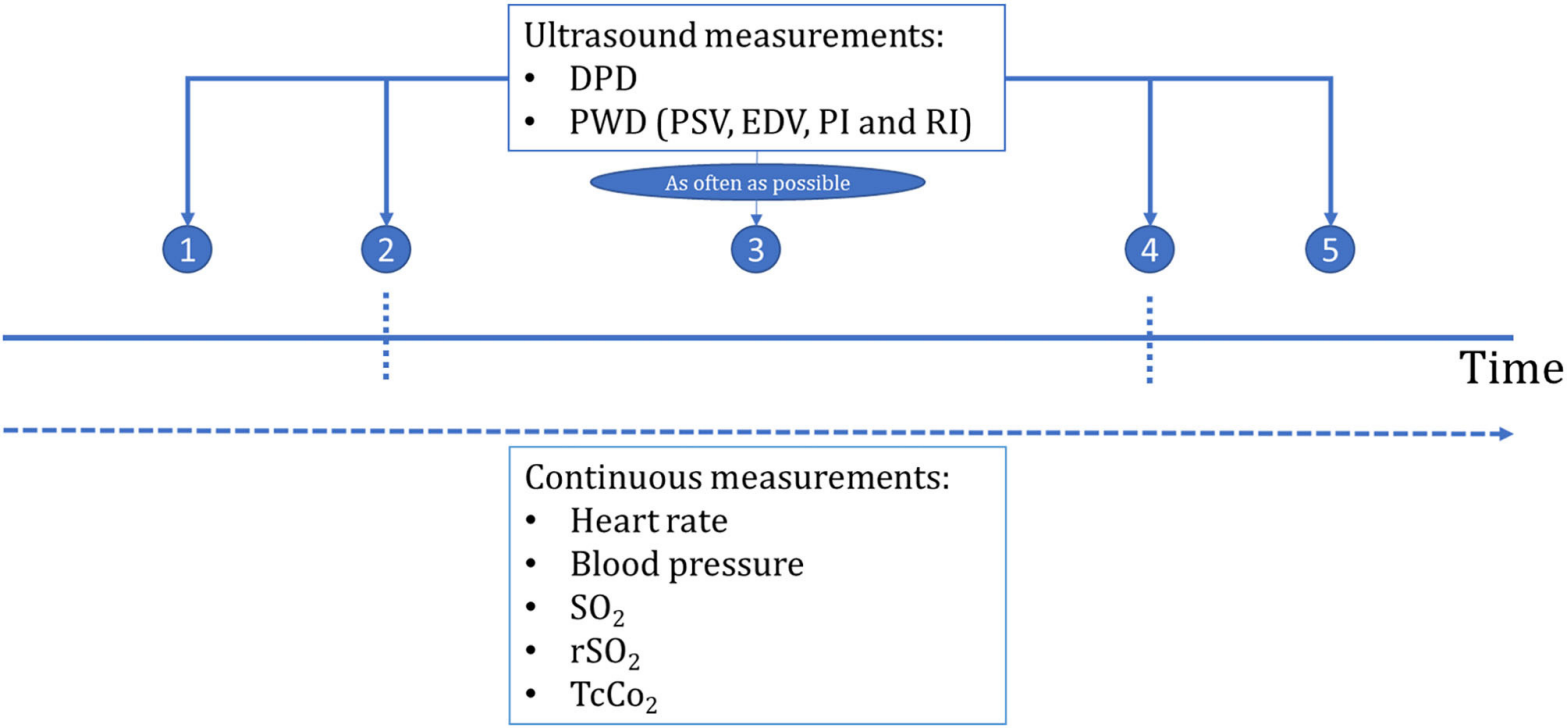
Overview of the measurements. Description of timepoints on the horizontal axes: period 1, at PICU preoperative; 2, after induction of anesthesia before surgery; 3, during surgical procedure; 4, after completion of the surgery and before transport to PICU; 5, PICU post-operative; SpO_2_, oxygen saturation; rSO_2_, cerebral oxygenation; PSV, peak systolic velocity; EDV, end diastolic velocity; RI, the resistivity index; PI, pulsatility index.

Preoperative transfontanellar ultrasound with DPD and PWD measurement was obtained in the PICU within the hour before transport to the operation theater. Serial intraoperative measurements were performed as frequently as possible, without interfering with regular perioperative care. A final measurement was performed within 30 min after surgery in the PICU. A schematic overview of all measurements is provided in Figure 1.

### Ultrasound Protocol

A clinical ultrasound machine, Zonare ZS3 (Mindray Medical International, Hoevelaken, Netherlands) with a high frequency linear probe (L20-5) was used for all ultrasound measurements. DPD and PWD are standardly available and commonly used modes on ultrasound machines. The frequency for DPD was 11 MHz with a medium wall filter, the velocity scale was set as low as possible. For PWD, frequency was set to 8 MHz with a low wall filter.

**Figure 2:**
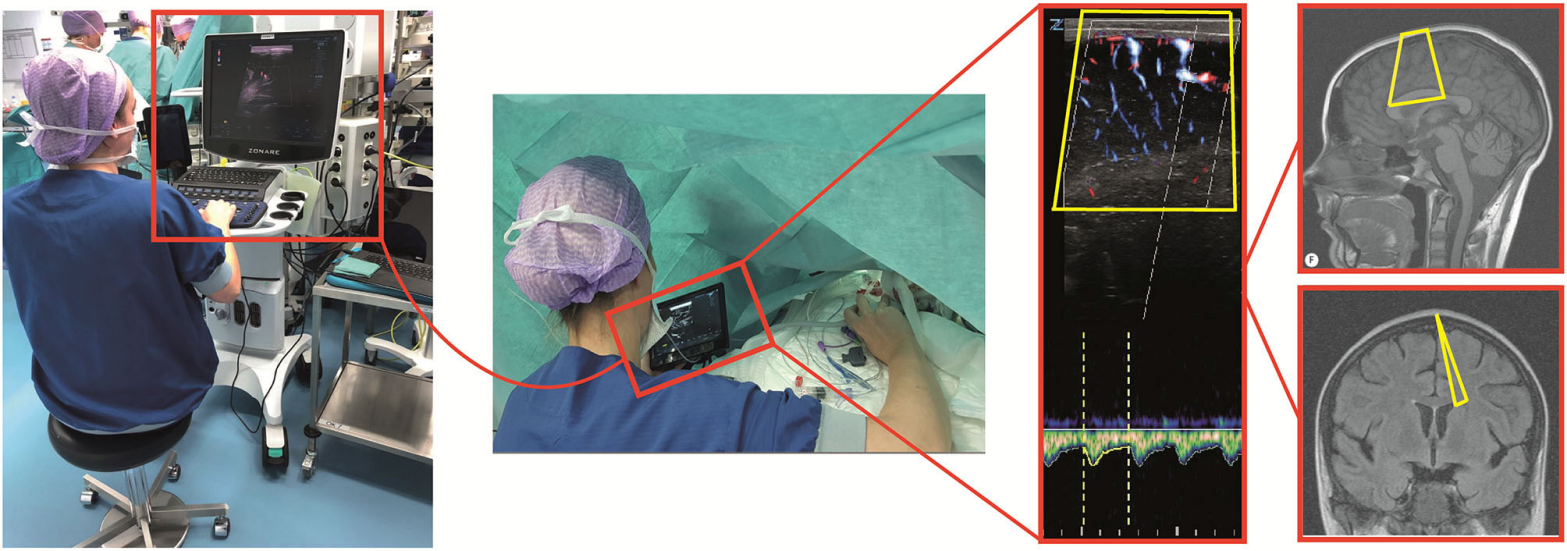
Ultrasound measurement setup. **(Left)** One researcher controlled the ultrasound machine outside the workspace of the surgeon and anaesthesiologist. **(Middle)** One researcher held the probe and performed the ultrasound scan with the feedback by an extended screen. **(Right)** The cerebral vasculature in the ultrasound plane was visualized with power Doppler. The spectral Doppler gate was placed in a vertically oriented part of this pial artery.

Two researchers (SC, AK) were trained by a brain ultrasound specialist and neonatologist (PG) in performing transfontanellar ultrasound in neonates. For practical reasons and limited space available in the operating theater, two researchers were needed to perform measurements; one to control the ultrasound machine and one to hold the probe and perform the scan (Figure 2). Visual feedback of the scan for this second person was provided by a 9-inch extension screen connected to the ultrasound machine. This way the ultrasound machine was kept out of the workspace of surgeon and anesthesiologist.

Since the area adjacent to the lateral ventricle is one of the border zones prone for watershed injury (3, 4), the probe was placed sagittally on the anterior fontanel on the plane adjacent to one of the lateral ventricles. Then, the cerebral vasculature in the ultrasound plane was visualized with DPD. Measured cortical pial arteries were perfused by the anterior cerebral artery. A recognizable pial artery confirmed the plane location for subsequent measurements in the same patient. The spectral Doppler gate was placed vertically in the pial artery (Figure 2), and the beam-to-flow angle was corrected. In some patients, both the left and right hemisphere were measured during surgery. In others, it was not possible to find the same pial artery for every measurement. We only used measurements in the same pial artery in the same hemisphere to compare measurements. Peak systolic velocity (PSV) in cm s^−1^, end diastolic velocity (EDV) in cm s^−1^, resistivity index (RI), and pulsatility index (PI) values were collected from PWD. RI is an arterial resistivity index which quantifies pulsatile blood flow and reflects resistance of CBF (13, 14). RI is calculated by (PSV–EDV)/PSV. PI is the difference in pulsatile flow velocities divided by time-averaged velocity (15). PI is calculated by (PSV–EDV)/V_mean_ in which V_mean_ is the time-averaged velocity of the PWD in cm s^−1^. Even though the different parameters are interdependent, they provide complementary information: PSV and EDV reflect absolute flow velocities, while RI and PI reflect intra-beat ratios.

### Data Analysis

Data were collected at multiple time points; in the PICU preoperatively (period 1), after induction of anesthesia, before start of surgery (period 2), during surgical procedure (period 3), after surgical procedure (period 4), and after returning in the PICU post-operatively (period 5). Retrospectively, transfontanellar ultrasound measurements were analyzed off-line for changes in cortical CBFV in the perioperative period and for their relation to perioperative management by studying HR, MABP, oxygen saturation, rSO_2_, transcutaneous pCO_2_, and VIS in relation to PSV, EDV, RI, and PI. We presented visualized measurement series with the most repetitive measurements if the same pial artery was not identified.

Data were analyzed in absolute values for each time point. Data and graphs are expressed as medians with inter quartile ranges (IQR) and outliers [MathWorks MATLAB v9.7.0.1190202 (R2019b), Natick, Massachusetts, United States]. During surgical procedure, multiple measurements were obtained and presented as the medians of the mean values.

## Results

**Figure 3:**
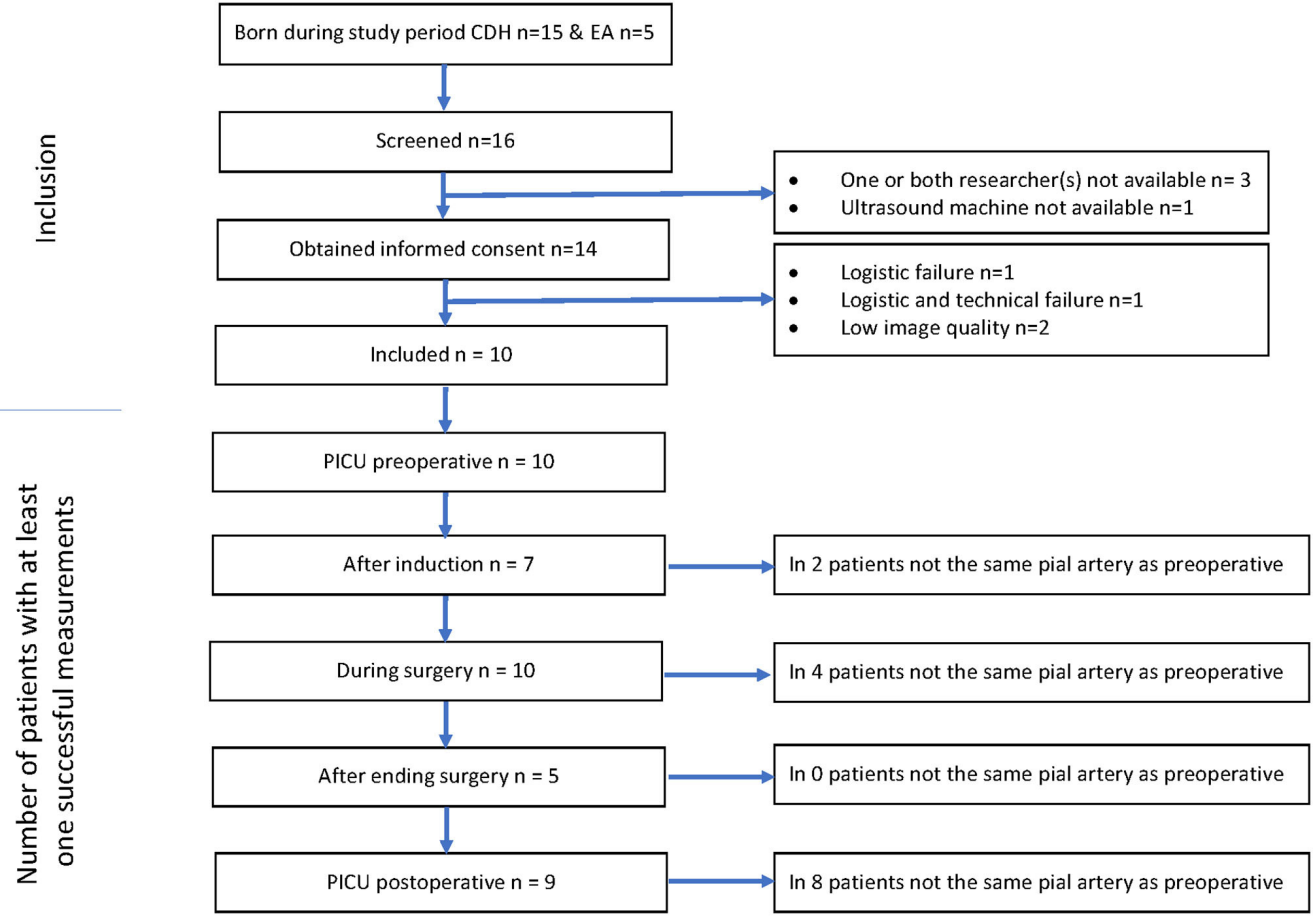
Inclusion and feasibility flowchart.

Informed consent was obtained for 14 neonates. In four patients it was not possible to obtain adequate measurements because of our learning process (*n* = 1), logistic and technical failure due to small operation theater (*n* = 1), the position of the surgeon near the neonate's head (*n* = 1), and insufficient image quality because of small fontanel size and too much hair (*n* = 1) (Figure 3). Data of 10 neonates were analyzed. Median gestational age was 39.4 weeks (IQR 38.4–40.5), birth weight 3,009 grams (IQR 2,500–3,234), age at surgery 2.5 days (IQR 2–3), and duration of surgical procedure 106 min (IQR 85–123). One patient had surgery in the PICU, the other nine patients had surgery in the operation theater (Table 1 and Appendix 1 in the Supplementary Material). Five patients were sedated and mechanically ventilated preoperatively. In all patients, routine cranial ultrasound examinations were performed 1 day before and after surgery and no signs of brain injury were found.

**Table 1:** Patient characteristics.

**Patient number**	**Anomaly**	**Sex**	**GA (weeks + days)**	**BW**	**Age at surgery (d)**	**Preoperative sedated and ventilated**	**Anesthesia intraoperative**	**Surgical approach**	**Duration surgery (min)**	**VIS-score PICU preoperative**	**Range VIS score intraoperative**	**VIS-score PICU post-operative**
1	CDH	M	38 + 5	3,000	12	Yes	Midazolam	Laparotomy	105	5	15	5
2	CDH	F	38 + 2	3,260	2	Yes	Sevoflurane	Laparotomy	76	0	1–12	3
3	CDH	F	41 + 0	3,420	2	No	Sevoflurane	Laparotomy	70	0	3–8	8
4	CDH	M	40 + 1	3,018	3	No	Sevoflurane	Thoracoscopy, conversion	99	0	6–56	8
5	CDH	M	40 + 4	3,155	3	Yes	Sevoflurane	Thoracoscopy	128	0	0–8	0
6	CDH	M	38 + 4	2,700	4	Yes	Sevoflurane	Thoracoscopy	106	27	17–27	15
7	CDH	F	40 + 6	4,042	3	Yes	Sevoflurane	Thoracoscopy, conversion	170	0	5–20	0
8	OA	M	40 + 2	2,970	2	No	Sevoflurane	Thoracoscopy	142	0	0–48	0
9	OA	M	34 +1	1,950	1	No	Sevoflurane	Thoracotomy	80	0	0	0
10	OA	M	32 + 6	1,734	1	No	Sevoflurane	Thoracotomy	108	0	0–10	0

*CDH, congenital diaphragmatic hernia; OA, esophageal atresia; M, male; F, female; GA, Gestational Age; BW, birth weight; Vasoactive-inotropic score (VIS): dopamine + dobutamine + 10x milrinone + 100x epinephrine + 100x norepinephrine + 1,000x vasopressin, all dosages in microgram per kilogram per minute (16)*.

### Feasibility

**Figure 4:**
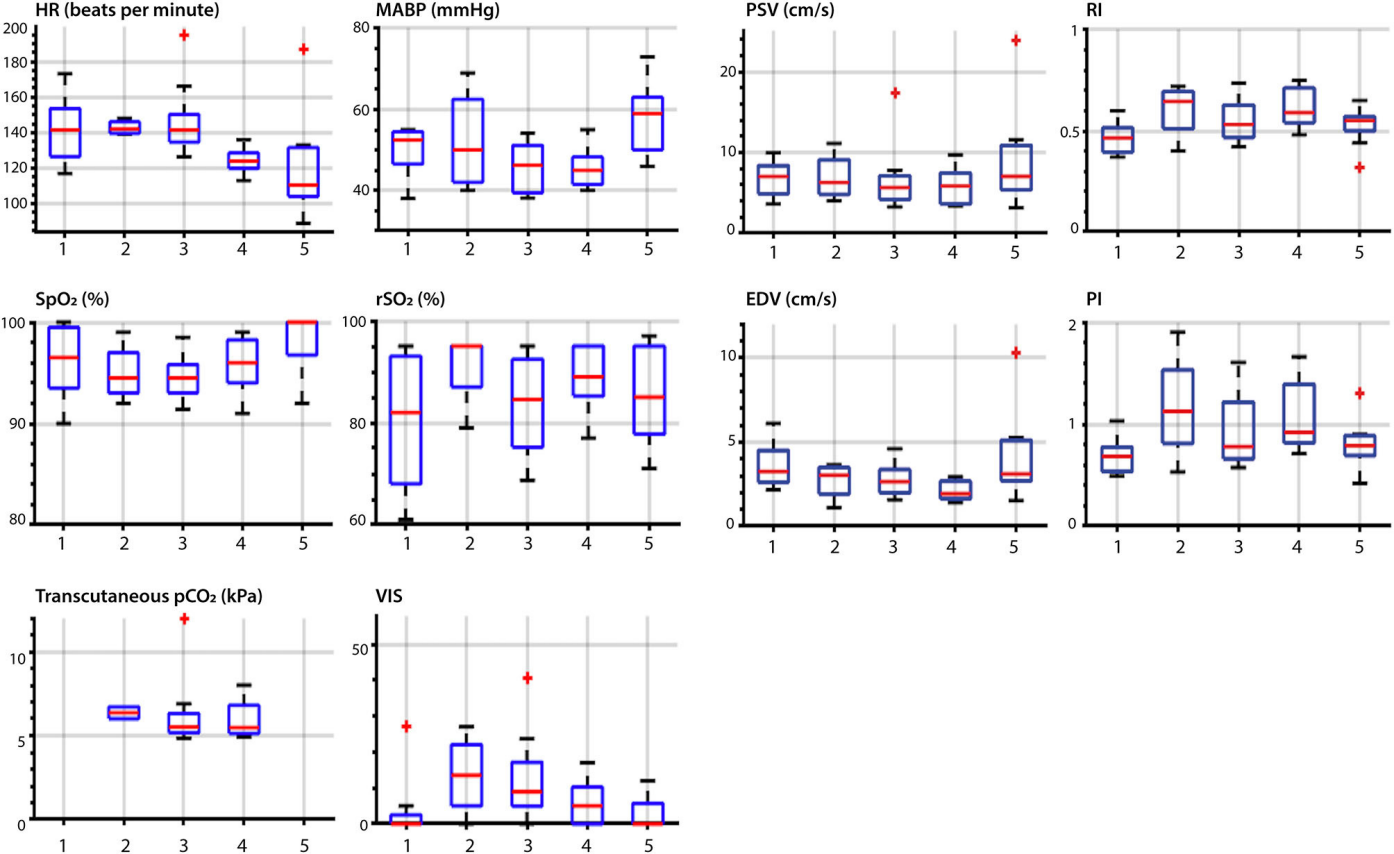
Overview of vital parameters and ultrasound parameters during the perioperative measurements. Description of timepoints on the horizontal axes: 1, at PICU preoperative; 2, after induction of anesthesia before surgery; 3, during surgical procedure; 4, after completion of the surgery and before transport to PICU; 5, PICU post-operative; HR, heart rate; MABP, mean arterial blood pressure; SpO_2_, oxygen saturation; rSO_2_, cerebral oxygenation; PSV, peak systolic velocity; EDV, end diastolic velocity; RI, the resistivity index; PI, pulsatility index. The red central mark of the box indicates the median and the bottom and top of the box indicates the interquartile ranges. The whiskers end at the most extreme data points not considered outliers, which are marked with a red +.

**Figure 5:**
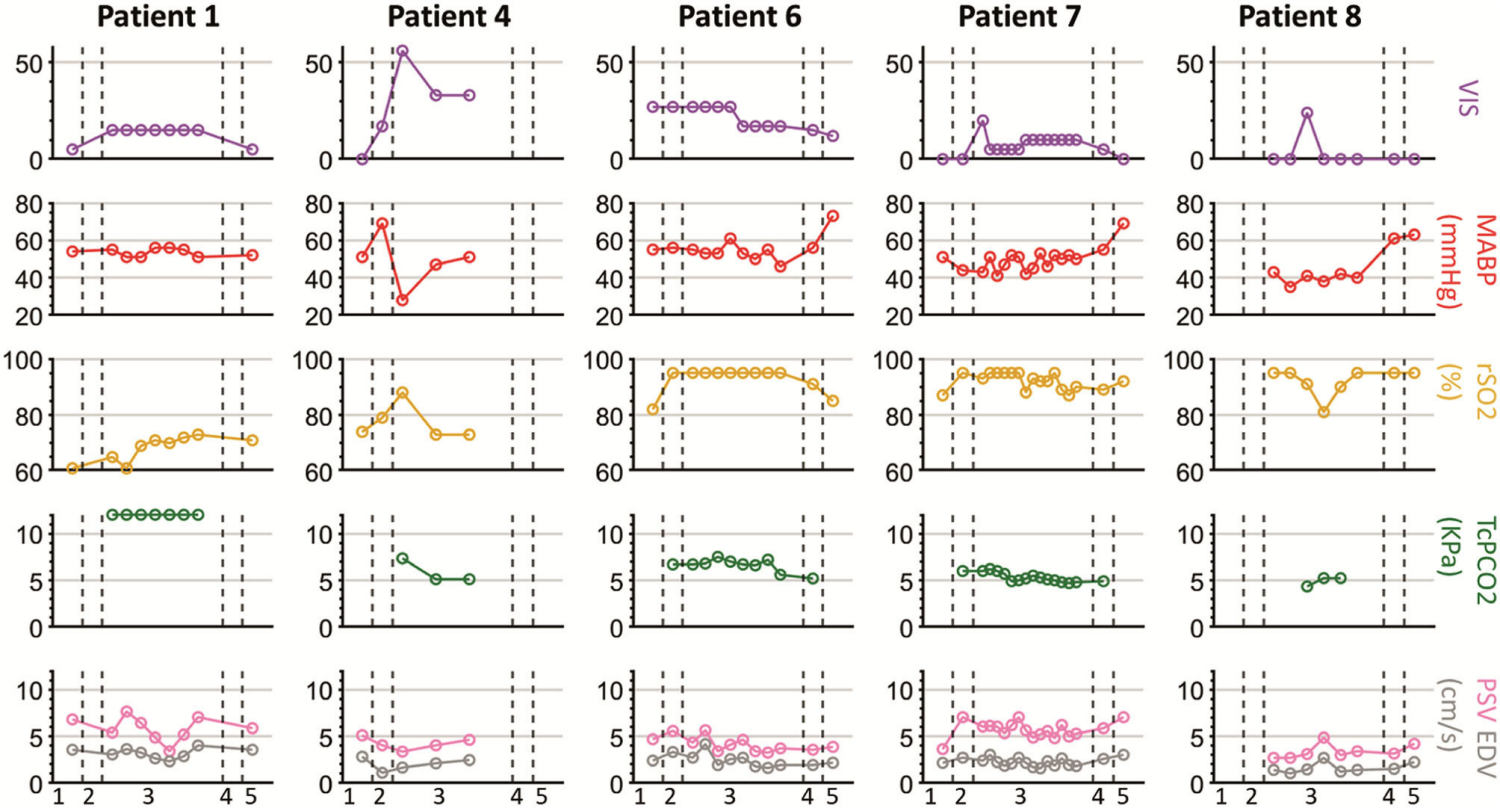
Measurements in patients during stable hemodynamic equilibrium with low VIS. Timepoint 1, at PICU preoperative; 2, after induction of anesthesia before surgery; 3, during surgical procedure; 4, after completion of the surgery and before transport to PICU; 5, PICU post-operative.

**Figure 6:**
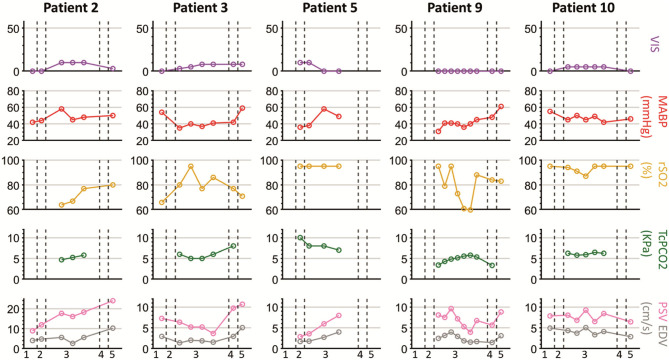
Measurements in patients during less stable hemodynamic equilibrium with high VIS. Timepoint 1, at PICU preoperative; 2, after induction of anesthesia before surgery; 3, during surgical procedure; 4, after completion of the surgery and before transport to PICU; 5, PICU post-operative.

Preoperative measurements were successfully performed in all 10 patients. It was possible to perform measurements after induction in seven patients. In five patients it was possible to measure the same pial artery. It was possible to perform measurements in all 10 patients during surgery. It was possible to follow up the same pial artery during surgery, although in four patients it was not the same artery as measured preoperatively. There were 4–14 successful ultrasound measurements per patient during surgery (Figures 4–6). It was possible to perform measurements after surgery in five patients in the same pial artery as during surgery. Post-operatively in the PICU, it was possible to perform measurements in nine patients. It was not possible to measure the same pial artery in one patient (Appendix 2 in the Supplementary Material). The research setup (Figure 2) with two persons led to efficient good-quality imaging without interfering with the anesthesiologist's or surgeon's workspace.

### Perioperative Measurements

Perioperative median HR ranged between 111 and 142 bpm with a decrease post-operatively (Figure 4). Median MABP ranged between 46 and 59 mmHg increasing post-operatively. Saturation remained above 90% in all patients during surgery and cerebral oxygenation increased after induction with a median up to 95%. Transcutaneous pCO_2_ was measured intraoperatively with a median varying between 5.5 and 6.3 kPa. There was little need for preoperative hemodynamic support, but support was needed intraoperatively (VIS 0-56, Table 1 and Appendix 3 in the Supplementary Material). Administered vasoactive and inotropic drugs were norepinephrine, epinephrine, dobutamine and milrinone. The highest VIS during an ultrasound measurement intraoperatively was 40 (Appendix 3 in the Supplementary Material).

Cortical CBFV changes occurred perioperatively as well. Median PSV ranged between 5.7 and 7.0 cm·s^−1^ and EDV between 1.9 and 3.2 cm·s^−1^. Median RI and PI increased over time; RI increased from 0.47 to 0.65 after induction and decreased intraoperatively to 0.53. PI increased from 0.69 to 1.1 after induction, decreased intraoperatively to 0.78 and post-operatively in the PICU to 0.78 (Figure 4).

### Cerebral Ultrasound Parameters in Relation to Perioperative Management

For the nine out of ten patients who were operated in the operation theater and anesthetized with sevoflurane, post-operative PSV and EDV were higher than intraoperatively. In patients with limited need of hemodynamic support (maximum VIS of 12 or deltas of <12), the trend of PSV and EDV corresponded with the MABP trend (patients 2, 3, 5, 9, 10) (Figure 5). In these five patients, especially the pattern and fluctuations of EDV were similar to those of MABP. In all patients, fluctuations in PSV were larger than fluctuations in EDV. Differences between PSV and EDV increased intraoperatively over time. A comparable trend between rSO_2_ and PSV/EDV was observed in two patients (patient 2, 9). rSO_2_ fluctuated more than PSV and EDV. In patients with higher need of hemodynamic support, PSV and EDV trends corresponded less with the MABP trend (patients 1, 4, 6, 7, 8). In these five patients VIS was not only higher (maximum VIS of 56), but also had larger changes in dosages (VIS ranging from 10 to 50) compared to the patients in whom PSV and EDV showed the same trend as MABP (Table 1). One patient (patient 7) had an increase in VIS of 20 which was reduced to a VIS of 5 at the start of the surgical procedure (Figure 6). No relation between MABP and PSV/EDV could be observed during a VIS of 20. Once VIS decreased and modifications in VIS remained limited, changes in PSV and EDV started showing the same trend as MABP. No comparable trend between rSO_2_ and PSV/EDV was observed in these five patients.

## Discussion

This study shows it was feasible to perform serial intraoperative transfontanellar ultrasound measurements of the pial arteries with DPD and PWD during high-risk non-cardiac neonatal surgery. In six out of ten patients, it was possible to perform repetitive measurements to obtain longitudinal perioperative cortical CBFV data. Furthermore, we observed large changes in cortical CBFV perioperatively which were independent of heart rate or measures of cerebral oxygenation, but did correspond with mean arterial blood pressure during limited need for hemodynamic support.

### Feasibility

Adaptability of surgeons and anesthesiologists was crucial in this feasibility study because we had to interfere with their workspaces. The setup (Figure 2) with two sonographers enabled us to obtain serial measurements with limited influence on perioperative care. The feasibility of repetitive measurement in the same pial artery in the same plane mainly depended on position of both the patient and the surgeon. When the neonate was positioned laterally for thoracotomy or thoracoscopy, there was very limited maneuvering space. During thoracoscopic repair the surgical workspace is more around the patient's head, making it especially difficult to perform transfontanellar ultrasound measurements without interfering with the surgical procedure.

In four of the fourteen patients it was not possible to perform ultrasound measurements or to obtain sufficient image quality. Measurements in the first patient failed because of technical issues, mainly related to our learning process. This was our first attempt to measure intraoperatively and we had to optimize the research set-up. It was challenging to position the ultrasound probe at the neonate's head, since the neonate was placed in the middle of the operation table. Additionally, the extended screen was placed behind the researcher. The lack of visual feedback resulted in insufficient image quality. Another patient was operated in a small operation theater with minimal space for our research set-up with an ultrasound machine and two researchers. Measurements could not be obtained without interfering with regular care. For one patient, the position of the surgeon was adjacent to the neonate's head, which made ultrasound measurements impossible without hindering the surgical procedure. Even though there were logistic and technical issues in measurements in three patients, only in one patient the measurements failed due to patient characteristics. This neonate had a small fontanel size and too much hair which resulted in insufficient image quality.

The amount of successful perioperative measurements varied between 4 and 14 measurements per patient. The main factors that explained this large variation are the learning curve and operation time. Over time it became easier to find the same pial artery intraoperatively, this resulted in a reduction in time for ultrasound scanning. Furthermore, measurements were obtained as often as possible, without interfering with the perioperative care. In longer surgeries, there were more opportunities for ultrasound scanning. However, multiple interventions by the anesthesiologist could be needed if a neonate became cardiorespiratory instable during the surgical procedure, which led to less available moments for ultrasound measurements.

Successful, repetitive measurements of the same pial artery preoperatively and intraoperatively was possible in 60% of the patients. Intraoperatively, repetitive measurements of the same pial artery were possible in 100% of the patients. This study shows that cortical CBFV changes intraoperatively, suggesting that monitoring pial artery perfusion provides crucial information about cortical CBFV, whether it is the same pial artery as measured preoperatively or not. Follow-up of the same pial artery perioperatively might be ideal, although one could argue if the preoperative clinical condition of these patients provides representative baseline measurements of cortical CBFV.

### Cerebral Ultrasound Parameters in Relation to Perioperative Management

Our study suggests that cortical CBFV changes in the perioperative period, while changes in the vital parameters remained limited to fluctuation in MABP and changes in PSV and EDV are related to changes in MABP, except when MABP was strongly manipulated with vasoactive and inotropic drugs. Limited fluctuations of hemodynamic support—maximum delta VIS of 12 or maximum VIS of 12—had no visible effect on the relations between MABP and PSV or EDV. Relations between the ultrasound parameters and rSO_2_ were not found.

Current techniques monitoring vital parameters (HR, MABP, SpO_2_, and rSO_2_) are poor approximations of brain perfusion in the neonate. Transfontanellar ultrasound measurements with DPD and PWD is a sensitive and objective method that might be of additional value in the perioperative period, especially during great need of hemodynamic support. One study proofed it is feasible to predict fluid responsiveness with transfontanellar ultrasound intraoperatively. Although this was measured in the internal carotid artery during cardiac surgery in older patient (9).

An earlier study of our team found that the maximum dose of vasoactive medication was negatively associated with verbal and visuospatial memory in 8-year-old survivors of neonatal extracorporeal membrane oxygenation and CDH survivors (1). The patients in our current study participate in a structured long-term follow-up program which could clarify the effect of these VIS fluctuations on brain development later in life.

### Regulation of Cerebral Blood Flow

Regulation of CBF in the healthy human brain is complex and multiple regulation systems are involved; cerebral autoregulation, flow-metabolism coupling, and neurogenic regulation (17). Cerebral autoregulation is affected by vasoactive and inotropic medication and PaO_2_ and PaCO_2_ have an effect on cerebral vascular resistance (5, 6, 18). Vasoactive and inotropic medication mediate changes in the cerebral vascular resistance by the cerebrovascular adrenergic receptors. In adults, the administration of norepinephrine, which was the most administered inotropic drug in our study, led to unchanged or decreased cortical CBFV, unchanged or increased cortical CBFV and an increased cerebral perfusion pressure (19–24). This may explain our incoherent results in cortical CBFV during changes in vasoactive and inotropic medication.

Neonates are prone to intraoperative hypercapnia (25–27). However, the effect of hypercapnia on cerebral perfusion in anesthetized neonates is virtually unknown (5). Research with transcranial Doppler ultrasound of the middle cerebral artery during hypercapnia in anesthetized adults showed increased PSV, EDV, and decreased RI and PI, whereas hypocapnia had the opposite effects (28). In our study, the variance of CO_2_ was limited and the number of other highly changing parameters was too big to observe an effect of changes in PaCO_2_.

Additionally, the CBF-metabolism coupling might be affected during anesthesia, when the cerebral activity is reduced (29). In this study, the PSV and EDV increased post-operatively in all patients anesthetized with sevoflurane intraoperatively. This suggests that the cerebral oxygen consumption increased post-operatively due to an increase in cerebral activity and metabolism. All of the above-mentioned changes might be determinants of the development of brain injury. Previous research showed that there is a link between fluctuation and extreme values in cortical CBFV and germinal matrix hemorrhages, intraventricular hemorrhages and white matter injury in (preterm) neonates (30–32). However, a correlation between Doppler parameters and structural brain damage has not yet been found, possibly explained by the lack of frequent measurements in previous research (33).

### Improvements

A possible improvement to increase continuity could be a probe holder designed for neonatal surgery, making the second sonographer redundant. This would also allow measuring at specific moments or during specific surgical interventions without interfering with the anesthesiology or surgery. A probe holder would solve the technical and logistical issues we encountered and increase the feasibility of the measurements. An example of transfontanellar Doppler ultrasound in neonates is the NeoDoppler probe, which enables measuring cerebral blood flow velocities in a cylindrical shape with a diameter of 1 cm and a depth between 3 and 35 mm (34). Another improvement could be made in optimizing vascular imaging. The ultrasound technique used in this study does not provide information about microcirculation in the (sub)cortex. A promising technique is high frame rate ultrasound (>1,000 frames per second), which enables more sensitive vascular imaging throughout the imaging plane and facilitates quantification in every vessel (35). In 2014, Demené et al. used high frame rate ultrasound for neonatal brain scanning for the first time, which resulted in high resolution images of the vascular network with simultaneous quantitative data of the ultrasound plane (36).

In the future, transfontanellar ultrasound measurements with DPD and PWD could guide clinicians in detecting fluctuations in CBFV. Potentially, this could aid in preventing brain injury by early detection of potential hazardous CBFV evoking protective intervention. Monitoring of the pial arteries could especially lead to a reduction in parenchymal lesions and supratentorial watershed infarction. The combination of DPD and PWD enables to image real-time cerebral perfusion. This allows for visualizing the vessel tree and monitoring CBFV throughout the surgery. Future additions of high frame rate ultrasound and the use of a probe holder would expand the possibilities of this technique.

In conclusion, this study shows that it is possible to perform transfontanellar ultrasound measurements in the pial arteries with DPD and PWD during major high-risk non-cardiac neonatal surgery. Cortical cerebral blood flow does fluctuate perioperatively, which could not be observed with the currently used monitoring techniques, especially not in neonates with higher need of hemodynamic support. Therefore, the next step would be to implement transfontanellar ultrasound with DPD and PWD in a larger group of patients perioperatively.

## Data Availability Statement

The raw data supporting the conclusions of this article will be made available by the authors, without undue reservation.

## Ethics Statement

The studies involving human participants were reviewed and approved by Medical Ethical Committee of Erasmus Medical Center. Written informed consent to participate in this study was provided by the participants' legal guardian/next of kin.

## Author Contributions

SC and AK: conception and design of the work, the acquisition, analysis and interpretation of data, drafting the work, and final approval. HV, JB, JG, DT, RW, and NJ: conception and design of the work, analysis and interpretation of data, revising it critically for important intellectual content, and final approval. PG: conception and design of the work, analysis and interpretation of data, revising it critically for important intellectual content, and final approval of the version to be published. All authors contributed to the article and approved the submitted version.

## Conflict of Interest

The authors declare that the research was conducted in the absence of any commercial or financial relationships that could be construed as a potential conflict of interest.
