# Supplementary material for: Feasibility of Doppler Ultrasound for Cortical Cerebral Blood Flow Velocity Monitoring During Major Non-cardiac Surgery of Newborns
Source: Front Pediatr. 2021 Mar 22;9:656806. doi: 10.3389/fped.2021.656806 (PMC8019737; doi:10.3389/fped.2021.656806)
Supplement: Supplementary file 1 [file Table_1.DOCX]

| Appendix 1. Additional patient characteristics | | | | |
| --- | --- | --- | --- | --- |
| Patient number | Anomaly | Side | Defect size/type | Liver |
| 1 | CDH | Left | C | Down |
| 2 | CDH | Left | A | Down |
| 3 | CDH | Right | A | Up |
| 4 | CDH | Left | A | Down |
| 5 | CDH | Left | B | Down |
| 6 | CDH | Left | B | Down |
| 7 | CDH | Left | C | Down |
| 8 | OA |  | C+TEF |  |
| 9 | OA |  | C+TEF |  |
| 10 | OA |  | C+TEF |  |
