# Supplementary material for: Feasibility of Doppler Ultrasound for Cortical Cerebral Blood Flow Velocity Monitoring During Major Non-cardiac Surgery of Newborns
Source: Front Pediatr. 2021 Mar 22;9:656806. doi: 10.3389/fped.2021.656806 (PMC8019737; doi:10.3389/fped.2021.656806)
Supplement: Supplementary file 2 [file Table_2.DOCX]

| Appendix 2. Overview of all performed measurements | | | | | | |
| --- | --- | --- | --- | --- | --- | --- |
| Patient number | Anomaly | PICU preoperative (1) | After induction (2) | During surgery (3) | After ending surgery (4) | PICU postoperative (5) |
| 1 | CDH | 1 right | - | 7 right | 1 right |  |
| 2 | CDH | 1 left/1 right | 1 left | 2 left/3 right |  | 1 left/1 right |
| 3 | CDH | 1 left/ 1 right | - | 4 left | 1 left | 1 left |
| 4 | CDH | 1 right | 1 right | 3 right/2 right different pial artery | - | 1 right same different pial artery as during surgery |
| 5 | CDH | 1 left/1 right | 1 right/1 right in a different pial artery | 3 right in a different pial artery/2 left | - | 1 right |
| 6 | CDH | 1 right | 1 right | 7 right | 1 right | 1 right |
| 7 | CDH | 1 right | 1 right | 14 right | 1 right | 1 right |
| 8 | OA | 1 left | 1 left | 1 left/6 left in a different pial artery | - | 2 left same different pial artery as during surgery |
| 9 | OA | 1 left/1 right | - | 6 left in a different pial artery | 1 left same different pial artery as during surgery | 1 left same different pial artery as during surgery |
| 10 | OA | 1 left | 1 left different artery | 5 left | - | 1 left |
