# Supplementary material for: Feasibility of Doppler Ultrasound for Cortical Cerebral Blood Flow Velocity Monitoring During Major Non-cardiac Surgery of Newborns
Source: Front Pediatr. 2021 Mar 22;9:656806. doi: 10.3389/fped.2021.656806 (PMC8019737; doi:10.3389/fped.2021.656806)
Supplement: Supplementary file 3 [file Table_3.DOCX]

| Appendix 3. Overview of administrated vasoactive and inotropic drug in the perioperative period | | | | | | | |
| --- | --- | --- | --- | --- | --- | --- | --- |
| **Anomaly** | **Patient** | **PICU preoperative (1)** | **After induction (2)** | **During surgery (3)** | **After ending surgery (4)** | **PICU postoperative (5)** |  |
| CDH | 1 | Milrinone 0.5γ | Milrinone 0.5γ and start epinephrine 0.05γ and norepinephrine 0.05γ | Milrinone 0.5γ, epinephrine 0.05γ and norepinephrine 0.05γ | Milrinone 0.5γ and epinephrine 0.05γ. Stop norepinephrine 0.05γ | Milrinone 0.5γ |  |
|  | 2 | 0 | Start norepinephrine 0.12γ | ↓ norepinephrine to 0.10γ | Norepinephrine 0.10γ | ↓ norepinephrine to 0.03γ |  |
|  | 3 | 0 | Start norepinephrine 0.03γ | ↑ norepinephrine to 0.08γ | Norepinephrine 0.08γ | Norepinephrine 0.08γ |  |
|  | 4 | 0 | Start norepinephrine 0.17γ | ↑ norepinephrine to 0.56γ and  ↓ norepinephrine to 0.33γ | ↓ norepinephrine 0.11γ | ↓ norepinephrine 0.08γ |  |
|  | 5 | 0 | Start dobutamine 5γ and  epinephrine 0.05γ | Stop dobutamine 5γ and epinephrine 0.05γ | 000 | 0 |  |
|  | 6 | Milrinone 0.5γ, norepinephrine 0.12γ, epinephrine 0.1γ | Milrinone 0.5γ, norepinephrine 0.12γ, epinephrine 0.1γ | Milrinone 0.5γ, norepinephrine 0.12γ,  stop epinephrine 0.1γ | Milrinone 0.5γ, norepinephrine 0.12γ | Milrinone 0.5γ and  ↓ norepinephrine to 0.07γ |  |
|  | 7 | 0 | 0 | Start norepinephrine 0.2γ and  ↓ norepinephrine to 0.05γ and  ↑ norepinephrine to 0.1γ | ↓ norepinephrine to 0.05γ | Stop norepinephrine 0.05γ |  |
| OA | 8 | 0 | 0 | Start norepinephrine 0.48γ and  ↓ norepinephrine to 0.24γ and  stop norepinephrine | 0 | 0 |  |
|  | 9 | 0 | 0 | 0 | 0 | 0 |  |
|  | 10 |  | Start norepinephrine 0.05γ | Norepinephrine 0.05γ | Stop norepinephrine 0.05γ | 0 |  |
| γ: microgram per kilogram per minute | | | | | | | |
